# Evaluation of the Cytotoxicity of a Newly Developed Obturating Material for Pulpectomy in Primary Teeth Using Embryonic Toxicology, Brine Shrimp Lethality, and MTT Assay: An *In Vitro* Study

**DOI:** 10.1055/s-0045-1802571

**Published:** 2025-03-12

**Authors:** Noor Fathima J, Lavanya Govindaraju, Ganesh Jeevanandan, Prabhadevi C. Maganur, Satish Vishwanathaiah, Ali Ahmed Assiry, Ather Ahmed Syed

**Affiliations:** 1 Department of Pedodontics and Preventive Dentistry, Saveetha Dental College and Hospitals, Saveetha Institute of Medical and Technical Sciences, Saveetha University, Chennai, Tamil Nadu, India; 2 Division of Pediatric Dentistry, Department of Preventive Dental Sciences, College of Dentistry, Jazan University, Jazan, Saudi Arabia; 3 Department of Preventive Dental Science, Faculty of Dentistry, Najran University, Najran, Saudi Arabia

**Keywords:** cytotoxicity, MTT assay, obturation, primary teeth, pulpectomy

## Abstract

**Objective:**

The search for an ideal obturating material has taken precedence due to the revolution of the materials used in pediatric endodontics. With zinc oxide, calcium hydroxide, and metronidazole as its core constituents, an unique obturating material was developed. To aid in the healing process, the material should be biocompatible and most importantly it should not have any negative consequences. Thus, using embryonic toxicology, brine shrimp lethality, and methylthiazol tetrazolium (MTT) assay, the current investigation sought to determine the cytotoxicity of the newly developed obturating material, which contained calcium hydroxide, zinc oxide, and metronidazole at 2% 60–40 concentrations.

**Materials and Methods:**

In zebrafish embryonic toxicology method and brine shrimp lethality assay, five distinct concentrations of the new obturating material was tested and compared with the control in a 24-well plate containing fertilized zebrafish eggs and in 6-well plate containing nauplii, respectively. The hatching and the viability rate of the zebrafish embryos and survival rate of nauplii were calculated. In MTT assay, the percentage of fibroblast cell viability and the cell morphology was documented. A statistical analysis was performed on all of the collected data.

**Results:**

The hatching and the viability rate of the zebrafish embryos falls as the concentration of the new obturating material rises. The survival rate of the nauplii also falls with rise in the concentration of the obturating material. No cytotoxic effect was demonstrated by the novel obturating material on the human gingival fibroblasts up to 200 μg/mL concentration.

**Conclusion:**

The novel obturating material exhibits minimal cytotoxic effects even at increased concentrations.

## Introduction

The field of pediatric endodontics has revolutionized the dental treatment for children by effectively retaining the infected primary teeth in an asymptomatic condition until their natural exfoliation. Since then, the materials, instruments, and the techniques used in pediatric endodontics has undergone a radical transformation. 1 2 3 4 The key factor that decides the outcome of an endodontic therapy in deciduous teeth is thorough debridement and disinfection of the primary root canals. Even with sufficient chemomechanical preparation, some of the microbes get retained in the primary root canals due to their intricate and tortuous configuration leading to secondary infections and failure of the endodontic treatment. 5 6 The only agent that can suppress these trapped microbes is the obturating material used. The focus was thus directed toward identifying the ideal obturating material with maximum antibacterial activity for primary teeth, which is again a challenge as the obturating material despite being antimicrobial, it should also resorb at the same pace as that of the physiological resorption of the primary roots. 7 8

In pediatric dentistry, the two most used obturating materials are the combinations of zinc oxide and eugenol and calcium hydroxide and iodoform. Nevertheless, the studies have revealed that the possibility of intracanal resorption as the major drawback of the combination of Ca(OH)2 and iodoform and the eugenol component of the combination of zinc oxide and eugenol has been identified as an irritant to periapical tissues causing necrosis of the cementum and bone. 9 10 11 Thus, a pursuit for an ideal obturating material in primary teeth persists as there are no obturating materials reported till date as ideal for use in primary teeth.

Zinc oxide, calcium hydroxide, and metronidazole were the main ingredients used for the creation of a new obturating material used in this study. Based on the antimicrobial efficacy and resorption rate of the core components, the novel obturating material was developed in 12 different concentrations and was subjected to antimicrobial test against *Streptococcus* *mutans* and *Enterococcus* *faecalis* to determine the composition with highest effectiveness. Of which 2% 60–40 concentration of the new obturating material with metronidazole, calcium hydroxide, and zinc oxide, respectively, ascertained to be more efficient. 12 13 14 15

Evaluation of the cytotoxicity of the newly developed obturating material is of utmost importance, as the cytotoxic materials have the potential to kill the periapical cells. 16 Furthermore, for a material to be used in primary teeth, it is essential to be extra cautious as the developing permanent tooth bud lies just beneath the primary teeth. In case of extrusion of the obturating material, it is crucial that the material should be biocompatible to promote the healing process and above all, it should not have any adverse effects. Hence, the purpose of this study was to evaluate the cytotoxicity of the novel obturating material developed at 2% 60–40 concentration of metronidazole, calcium hydroxide, and zinc oxide on the embryonic zebrafish, nauplii shrimp, and human gingival fibroblastic cells using embryonic toxicology, brine shrimp lethality, and methylthiazol tetrazolium (MTT) assay.

## Materials and Method

### Study Design and Ethical Approval

The research was intended to be conducted *in vitro* to evaluate the cytotoxicity of the newly developed obturating material on the embryonic zebrafish, brine shrimp lethality, and human gingival fibroblasts. Institutional Review Board granted the ethical approval prior to the study's commencement (IHEC/SDC/PEDO-2125/21/537).

### Preparation of the Obturating Material

Note that 600 mg of calcium hydroxide was mixed with 400 mg of zinc oxide in powder form, to create 60–40 concentration, which was then kept in magnetic stirrer for 60 minutes to allow uniform and thorough mixture. With the aid of a mortar and pestle, 400 mg of metronidazole tablets were ground into powder. By combining 196 mg of the calcium hydroxide-zinc oxide mixture with 4 mg of metronidazole powder, a 2% 60–40 concentration of the new obturating material was created. 17

### Zebrafish Embryonic Toxicology

The cytotoxicity of the newly developed obturating material was tested using zebrafish ( *Danio rerio* ), which was procured from the Kolathur fish farm and was conducted in the Department of Pharmacology, SD College and Hospitals, Chennai, Tamil Nadu, India. A total of 10 females and 15 males were housed in distinct tanks. The tanks with the specimens were exposed to 14 and 10 hours of light and dark cycles, respectively. The temperature of the tanks and the pH of the water were regularly monitored so that a constant temperature of 28°C and pH within the range of 6.8 to 8.5 was maintained. The specimens were administered with piscine diet twice daily, which is a combination of shrimp and dry flakes. A transparent partition was introduced between the males and the females for nocturnal separation, which was removed in the subsequent mornings to facilitate reproduction to generate viable zebrafish eggs. The generated eggs of the zebrafish were carefully recovered and cleaned with E3 medium.

Following fertilization, the eggs were incubated in 24-well U-shaped culture plates. Each of the well contained 3 mL of E3 medium with 100 mg/L of standardized TiO2 contrast solution. Five different concentrations of the novel obturating material (5, 10, 20, 40, and 80 µg/mL) was tested and compared with the control, which comprised of the zebrafish eggs in E3 medium only. After 24 hours, the embryos' hatching and viability rates were recorded. The number of hatched embryos among the five embryos cultured in each well is known as the hatching rate, and the number of hatched embryos that were alive among the five embryos cultured in each well is known as the viability rate.

### Brine Shrimp Lethality Assay

A tank with proper aeration was set to which 1 L of distilled water and 30 g of iodine-free salt was added. One gram of *Artemia salina* eggs were then added to the tank and were left undisturbed for 24 hours to facilitate incubation. The next day, saline water was prepared by dissolving 2 g of iodine-free salt in 200 mL of distilled water and 10 mL of it was added to a 6-well enzyme-linked immunosorbent assay (ELISA) plates. Following which, nauplii were collected from the setup tank and five were added to each well. Five different concentrations of the novel obturating material (5, 10, 20, 40, and 80 µg/mL) were prepared and added to each well. The sixth well served as a control where nauplii were added to saline water only. The ELISA plates were then incubated for 24 hours, following which the number of dead and live nauplii in each well was counted manually. The counts were recorded and the percentage of live nauplii was calculated by dividing the number of dead nauplii by the number of total nauplii and multiplying with 100.

### MTT Assay

The human gingival tissue collection method was approved by the Human Ethical Committee of Saveetha University. Consent form was explained and signed by the patients prior to tissue collection. The tissue processing was done in a biosafety cabinet following strict sterilization protocols. Periodontal ligament tissues were collected from the interdental papilla of the healthy adolescents during the extraction of the premolars for orthodontic treatment. Note that 20 to 50 mg of the tissues were weighed and stored in sterile saline for 1 hour following which the gingival tissues were washed with phosphate-buffer saline (PBS) for 10 times to dilute the oral bacterial flora present on the collected tissues. The tissues were then sliced into fragments sized 1 to 2 mm using a surgical blade on a sterile petri plate containing the culture media. After being plated onto 25 cm tissue culture flasks, the human gingival tissue was kept undisturbed at 37°C in a humidified incubator with 5% CO _2_ for 48 hours. After 48 hours, the medium was switched. The cells were multiplied until there were enough of them to carry out experiments.

The human gingival fibroblast cells were plated separately in 96-well plate with a concentration of 5 ×10 ^3^ cells/well for indirect method and 5 × 10 ^4^ cells/well seeded on the top of the test materials for direct MTT assay in Dulbecco's modified Eagle medium media with 1× antibiotic solution and 10% fetal bovine serum (Gibco) in CO _2_ incubator at 37°C with 5% CO _2_ . The cells were washed with 100 μL of 1× PBS, then the cells were treated with novel obturating material (25–400 μg/mL) and incubated in CO _2_ incubator at 37°C with 5% CO _2_ for 24 hours. When the treatment period came to an end, the medium was aspirated from the cells. A CO _2_ incubator was used to incubate the 0.5-mg/mL MTT prepared in 1× PBS for 4 hours at 37°C. Following the incubation period, the MTT-containing medium was removed from the cells and cleaned with 100 μL of PBS. Once the crystals were formed, they were thoroughly mixed and dissolved in 100 μL of dimethyl sulfoxide. At 570 nm, the color's development was measured. The formazan dye becomes purple-blue in hue. Using a microplate reader, the absorbance was determined at 570 nm. The percentage cell viability was measured using the following formula: cell viability = [optical density (OD) of treated cells/OD of control cells] × 100. The cells were treated with and without novel obturating material (100, 200, and 300 μg/mL) for 24 hours' time point. After the treatment period, the cells were washed with PBS and observed in inverted phase contrast microscope to assess the cell morphology.

## Results

**Fig. 1:**
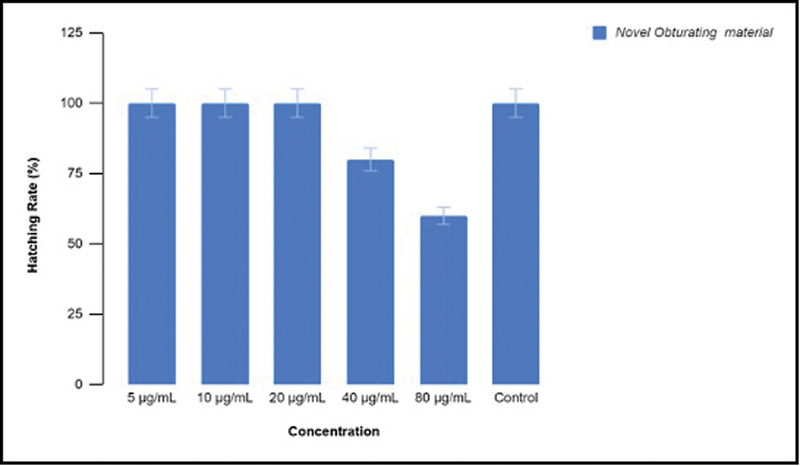
Hatching rate of the zebrafish embryos in different concentrations of the novel obturating material.

**Fig. 2:**
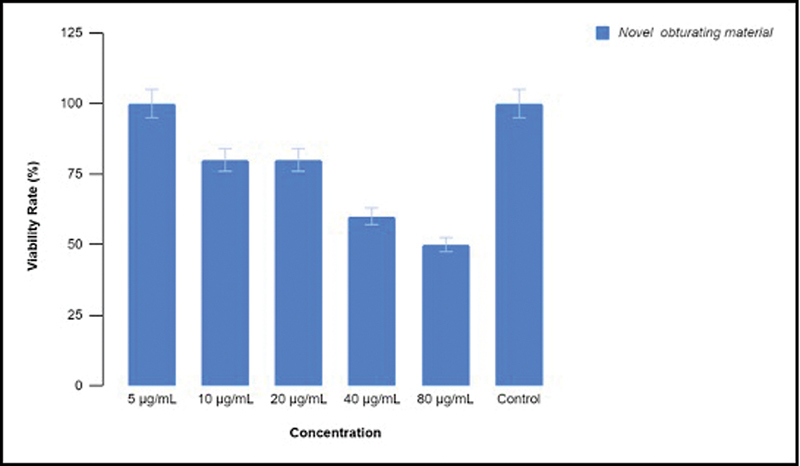
Viability rate of the zebrafish embryos in different concentrations of the novel obturating material.

Zebrafish embryonic toxicology study reveals that at concentrations of 5, 10, and 20 µg/mL of the new obturating material, the hatching rate of the embryos was 100%; however, as the concentration of the obturating material increased, the hatching rate decreased as depicted in Fig. 1 . The viability rate was also more in lower concentrations and showed a descending trend with increase in concentration ( Fig. 2 ).

**Fig. 3:**
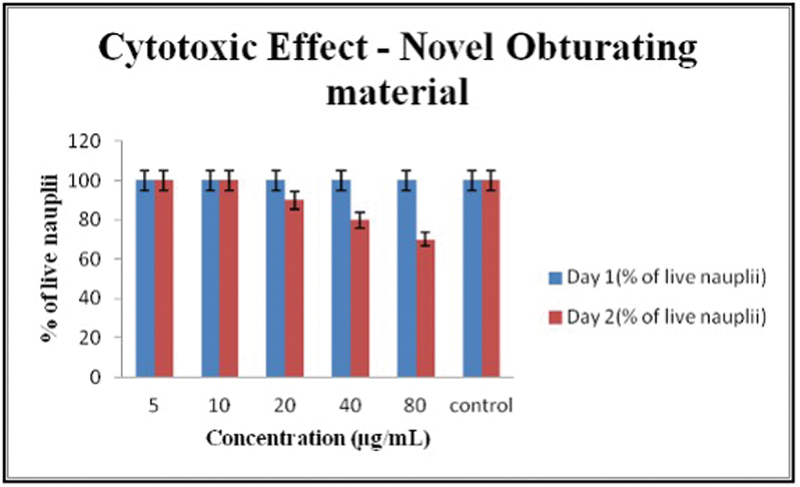
Survival rate of nauplii in different concentrations of the novel obturating material.

Brine shrimp lethality assay reveals that in day 1, the percentage of live nauplii was 100% at all the different concentrations of the novel obturating material and at day 2, the survival rate of nauplii decreased with increase in concentration ( Fig. 3 ).

**Fig. 4:**
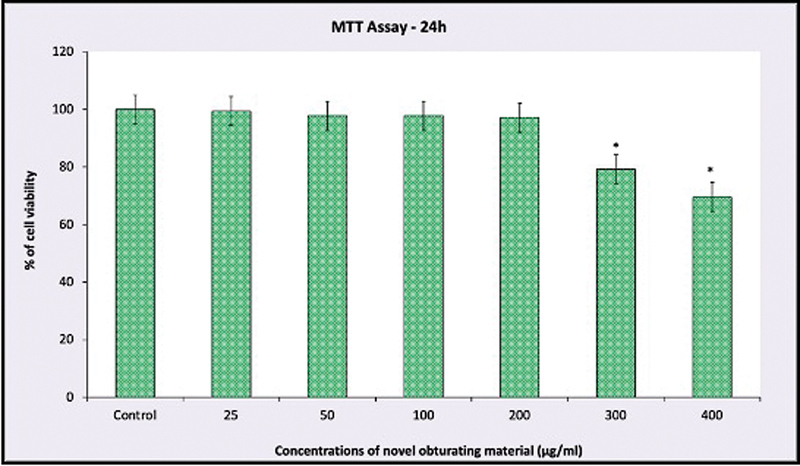
The cytotoxic effects of novel obturating material on human gingival fibroblast cells.

**Fig. 5:**
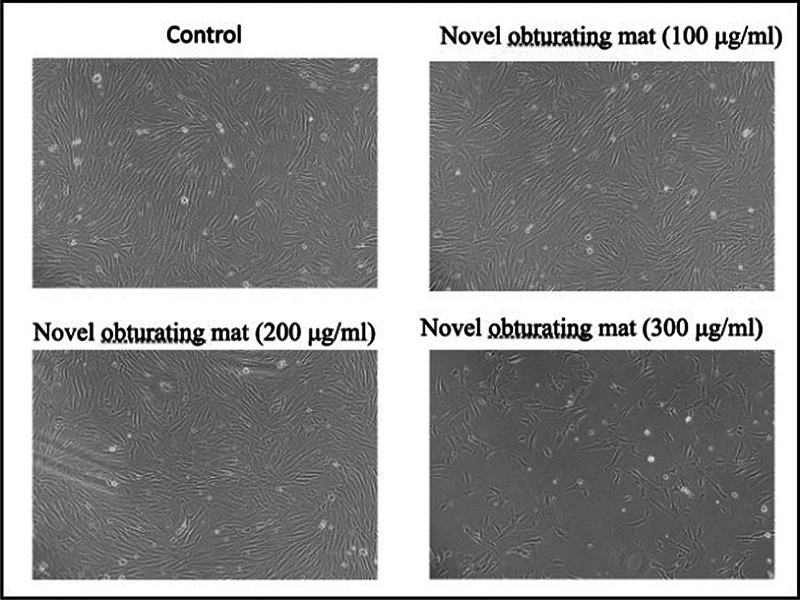
Effect of novel obturating material on cell morphology of human gingival fibroblast cells, observed under inverted phase contrast microscope.

Evaluation of the viability of the fibroblast cells using the MTT assay reveals that the obturating material shows no cytotoxic effect on the human gingival fibroblasts up to 200 μg/mL concentration of the obturating material. A significant difference was noted only at a concentration of 400 μg/mL of the obturating material ( Table 1 and Fig. 4 ). The morphology of the fibroblast cells when viewed under inverted phase contrast microscope shows that the cells are viable up to 200 μg/mL concentration of the obturating material ( Fig. 5 ).

**Table 1:** Evaluation of the cell viability using MTT assay

	Control	25 μg/mL	50 μg/mL	100 μg/mL	200 μg/mL	300 μg/mL	400 μg/mL
Mean	100	99.41	97.79	97.74	97.15	79.24	69.56
SE	0	12.14	11.9	11.16	12.22	12.75	9.11
*p* -Value		0.415	0.345	0.329	0.312	0.061	0.023 a

Abbreviations: MTT, methylthiazol tetrazolium; SE, standard error.

a Compared with the control group, *p* < 0.05.

## Discussion

The most important element influencing the outcome of endodontic therapy in primary teeth is the obturating materials. Obturating materials are expected to be confined within the root canal space; however, due to the morphology of the primary teeth and the physiological resorption, the material may get inadvertently extruded into the periapical area. 18 Such extruded material should not cause any tissue irritation or intervene with the process of healing. Hence, assessing the biological properties of the novel obturating material is essential to support its use in deciduous teeth.

In the present study, the cytotoxicity of the new root canal filling material for primary teeth was assessed using zebrafish embryonic toxicology study. In the past few decades, zebrafish have become an important model organism to examine the human diseases. The hatching rate and the viability rate of the zebrafish embryos show a descending trend with increase in the concentration of the new obturating material. The focus on zebrafish embryos in specific may limit the applicability of its findings to a wider variety of organisms, and hence, brine shrimp lethality assay was also conducted to obtain more conclusive results. The results of brine shrimp lethality assay were also in accordance with the zebrafish embryonic toxicology study. The new material developed for obturation in the primary teeth exhibited minimal cytotoxic effects with increase in the concentration of the material.

The study was further extended to evaluate the interaction of the newly developed material with the fibroblasts, as the fibroblastic cells make up the majority of the connective tissue, periodontal ligament, and are primarily present in the periapical regions. The MTT assay was used in this investigation to assess the cytotoxicity of the novel obturating material because it is known to be a simple and uncomplicated method to perform. Additionally, the findings were obtained more quickly, precisely, and consistently. MTT assay indicates the viability of the cell by converting the water-soluble MTT to insoluble purple formazan in the mitochondria of the living cells. 19 The results of the MTT assay in the present study show that the novel obturating material, which is a combination of zinc oxide, calcium hydroxide, and metronidazole, shows no cytotoxic effect on the fibroblasts till 200 μg/mL concentration. Even at 300 μg/mL concentration, the new material shows no statistically significant effect on the fibroblasts as compared with the control group.

As described by Dahl et al, 20 the cytotoxicity of the new material developed for obturation in primary teeth when rated based on the cell viability relative to the respective controls shows that the material is only slightly cytotoxic as 60 to 90% of the zebrafish, nauplii, and fibroblastic cells remained viable. And noteworthy is the fact that the cytotoxic effects were seen only with the increase in the concentration.

Although the first line of tests that assessed the cytotoxicity of the newly developed obturating material showed that the novel obturating material is biocompatible, further comparative studies with existing materials are necessary to draw final conclusions. Since the MTT assay only evaluates cell viability, it is also necessary to compare the novel obturating materials with the current materials for the other biocompatibility factors, such as genotoxicity and inflammatory potential. Also, further studies should be performed comparing the antimicrobial properties of the novel obturating material with the existing materials in use. Further clinical trials are needed to validate this conclusion and put it into practice and also to assess other features like its resorption rate and biocompatibility.

## Conclusion

Within the limitations of the present study, the newly developed obturating material for primary teeth at 2% 60–40 concentration of metronidazole, calcium hydroxide, and zinc oxide shows minimal cytotoxicity even at increased concentrations on the embryonic zebrafish, nauplii shrimp, and human gingival fibroblastic cells.
